# Perceived Case Management Needs and Service Preferences of Frequent Emergency Department Users: Lessons Learned in a Large Urban Centre

**DOI:** 10.1371/journal.pone.0168782

**Published:** 2016-12-21

**Authors:** Deborah Kahan, Daniel Poremski, Deborah Wise-Harris, Daniel Pauly, Molyn Leszcz, Donald Wasylenki, Vicky Stergiopoulos

**Affiliations:** 1 Department of Psychiatry, University of Toronto, Toronto, Ontario, Canada; 2 Centre for Urban Health Solutions, St. Michael’s Hospital, Toronto, Ontario, Canada; 3 Institute of Mental Health, Singapore; 4 Centre for Addiction and Mental Health, Toronto, Ontario, Canada; TNO, NETHERLANDS

## Abstract

**Objectives:**

This study aimed to explore the service needs and preferences of frequent emergency department users with mental health and addictions concerns who participated in a brief intensive case management intervention.

**Methods:**

We conducted semi-structured individual interviews with 20 frequent emergency department users with mental health and addictions challenges, 13 service providers involved in the delivery of a brief case management intervention, and a focus group with intervention case managers. Thematic analysis was used to explore perceived service user profiles, service needs and preferences of care.

**Results:**

Service users experienced complex health and social needs and social isolation, while exhibiting resilience and the desire to contribute. They described multiple instances of stigmatization in interactions with healthcare professionals. Components of the brief intensive case management intervention perceived to be helpful included system navigation, advocacy, intermediation, and practical needs assistance. Frequent service users valued relational responsiveness, a non-judgmental stance, and a recovery orientation in case managers.

**Conclusion:**

Interventions for frequent service users in mental health may be enhanced by focusing on the engagement of formal and informal social supports, practical needs assistance, system navigation, advocacy and intermediation, and attention to the recovery goals of service users.

## Introduction

Emergency department (ED) overcrowding has become a concern across many jurisdictions [1], as studies have identified that a small number of individuals account for a disproportionately high number of ED visits [2]. Frequent ED use has been associated with a substantial burden of illness and socioeconomic disadvantage [2–5]. To address the needs of this heterogeneous frequent ED user population, various interventions have been described in the literature. Among them, case management has received extensive attention [3, 6–11].

Case management involves a “customized care process” supporting service users and their connection to available health and social services, with the goal of enhanced health and service use outcomes [6]. Research to date has not shown consistent reductions in ED use associated with case management interventions for frequent ED users [3, 6–11]. Furthermore, existing systematic reviews of case management interventions have included heterogeneous patient populations of frequent ED users, and various approaches to case management, rendering conclusions about the effectiveness of case management interventions for any specific population of frequent ED users difficult to establish [6, 9].

Researchers have thus become interested in identifying particular subgroups of frequent ED users, as there may be “sharp differences” that could inform better tailored interventions [2]. Patients with mental health and substance use challenges are a particular group of interest, as they are over-represented among frequent ED users, and may experience complex support needs that require specialized interventions [9].

Furthermore, the literature describing the frequent ED user population has been based in large part on survey data of demographic information, comorbid conditions, and insurance type [12]. There are few qualitative studies highlighting the service users’ actual perspectives, and even less is known of the experiences of mental health and addictions frequent ED users [12, 13]. Qualitative methodology can generated nuanced insights into service users’ experiences and their perspectives on implemented interventions [12, 14, 15]. This information could help inform the development of tailored interventions producing superior outcomes [16].

Better identifying the needs, challenges, and strengths of the mental health and addictions frequent ED user population as well as key elements of case management interventions designed to support them would be of interest to many jurisdictions facing similar challenges. This qualitative study, which explores service user and provider perspectives in the context of a brief intensive case management intervention, achieved twofold aims: first, to expose the complexity of needs of frequent ED users with mental health and addictions challenges in a large urban centre in Canada, and second, to identify key service ingredients addressing service user needs and preferences.

## Methods

The study sample was drawn from the treatment arm of a randomized controlled trial (RCT) examining the effectiveness of a brief intervention for frequent ED users with mental health and/or substance use challenges in Toronto, Ontario (ClinicalTrials.gov Identifier: NCT01622244. Registered 4 June 2012). This trial was conducted from November 2012 to September 2014. Briefly, participants (N = 166) were adults 18 years or older with five or more ED visits over the past year to one of 6 participating hospital EDs. At least one visit was for a mental health or substance use concern. Participants were randomized to the intervention or treatment as usual group, and followed for 12 months. Research ethics board approval was obtained from all 6 participating hospitals (St. Joseph’s Health Centre, University Health Network, Centre for Addiction and Mental Health, Sunnybrook Health Sciences Centre, St. Michael’s Hospital, and Toronto East General Hospital).

### The intervention

Coordinated Access to Care from Hospital Emergency Departments (CATCH-ED) is a brief intensive case management intervention designed to facilitate the connection of frequent ED users with mental health and/or addictions challenges to primary care and appropriate community services. The aim of the intervention is to reduce acute care utilization, and improve continuity of care and health outcomes for this population. The intervention involves immediate access to brief case management over 4–6 months, with rapid access to team-based primary care and peer support. The intervention was implemented in the EDs of five general acute care hospitals and one specialty mental health hospital in Toronto. It involves a partnership with four community health centres, one peer support agency, and three community mental health organizations that provide case managers to participating hospitals. During the study period there were four transitional case managers (TCMs), carrying a caseload of approximately 1:15 clients each. Five TCMs were involved over the course of the intervention due to staff turnover. The TCMs engaged service users in developing individualized care plans and connected them with long-term services and supports. The TCMs were trained and supervised by a program manager.

### Data collection and analysis

A sample of 20 service user participants were purposively drawn from the intervention arm of the trial and completed qualitative semi-structured interviews six months after study enrolment. The interviews were conducted between August to December 2013. Written consent was sought from all research participants.

A peer interviewer with lived experience of mental illness conducted the interviews, aided by a semi-structured interview guide (S1 File). Questions focused on participants’ experiences of the intervention, including perceived frequent ED user service needs and preferences. All service providers were invited to participate in individual interviews, including five CATCH-ED TCMs, two primary care physicians at community health centres, three community health centre counselors, and three managers at participating community mental health agencies. A focus group was also held with four of the five CATCH-ED TCMs. All participants gave written informed consent, and service user participants received a $50 honorarium and two tokens for public transportation in order to reduce participation barriers. The interviews ranged in length from 30–90 minutes and were audiotaped and transcribed verbatim.

The transcripts were analyzed using thematic analysis [17]. Two members of the research team coded three transcripts independently and then met to compare findings. Emerging themes were highlighted using an inductive process. Once coding consensus was reached, one member of the research team coded the remaining transcripts using NVivo 10 software. Similar codes were grouped into themes, supported by direct quotations from the transcripts. The analysis was further developed by five members of the research team (D.K, D.Po, D.Pa, D.W, V.S), drawing from the frequency of contributing codes to identify key themes.

## Results

### Mental health and addictions frequent ED users’ profiles

Several common *themes* emerged from service user and provider narratives, including *complex health and social needs*, experiences of *stigma and discrimination* in healthcare interactions, *social isolation*, as well as *resilience and desire for societal contribution*.

#### Complex health and social needs

Service users described *complex health needs* related to frequently co-occurring mental health, substance use, and physical health problems and illnesses. Service providers similarly described the “complexity” of this population’s health and social needs, necessitating involvement of multiple health professionals, as contributing to the perception of “severely challenging” patients (Source: Participant 202- Service Provider).

“…[B]ecause of that acquired brain injury…and the balance issues and…the allergies combined with, addictions…of course the ED visits is based on—how you know- all the things that happen with being hit by a truck is and I think it contributes to me being a frequent [ED user] and then on top of that I have gastro issues that are quite severe…”

(Source: Participant 100015-Service User).

*Childhood abuse*, *neglect and trauma* were also notable experiences in servicer users’ past. Service users associated their trauma histories with later mental health and addictions problems.

“…then the light bulb went on and I saw the connection between the abuse, resulting in the anxiety, resulting in the [substance] use, resulting in the depression and then the suicidal thoughts and actions”

(Source: Participant 100098-Service User).

*Complex social service needs* were another feature of this population. The most commonly cited service needs involved housing and legal issues. Housing issues included service users trying to find housing, being in danger of eviction, and living in squalid housing conditions. As one service user stated compellingly- “I literally live in the middle of death” (Source- Participant 100025- Service User).

Unresolved legal issues included criminal charges, insurance claims, and custody battles. The interplay between mental and physical health challenges, substance use, and socioeconomic disadvantage further complicated the tasks and roles of case managers:

“…this is an average combination of issues—it could be trauma from child sexual abuse as a young kid, followed along with alcohol or substance use …to a very dysfunctional family, to potential violence they faced …incarceration…to anxiety around crowds … lack of money because their [government] allowance was cut short or whatever…the fact that they’re going to be evicted from the housing they have or the fact that they are on the street and don’t have housing…one client who faces all of this, it’s like the equivalent of ten clients”

(Source- Participant 202-Service Provider).

Although the majority of service users identified unmet support needs, a small number stated that they already had access to needed services and supports. For example, one service user discussed continued use of the ED despite having supports in place due to ongoing issues with pain, depression and anxiety.

“…for the issues that I have there wasn’t really like much that could be done, like I was already on social assistance in terms of financial, I was already on waiting lists for housing that I couldn’t really take advantage of, I was already seeing somebody for my psychology issues…, I already had a pain doctor like even though things were difficult there was not much more I feel like she could really assist me with”

(Source: Participant 10001- Service User).

#### Stigma and discrimination in healthcare settings

Frequent ED users described feeling dismissed, misdiagnosed, and generally unwelcome during healthcare encounters. Service providers similarly reported that health professionals harbored negative attitudes towards frequent ED users, and commented on mental health and addictions concerns being taken less seriously compared to physical health concerns in the ED setting.

“They have gotten that message from the system, right, so ‘You’re irritating, you’re obnoxious, you’re sucking up taxpayer funds,’ people have literally been told this to their face. You don’t need help, you have no business here, you are absolutely a nuisance. That’s our profession speaking to people, that’s not the general public that’s our actual profession… I find it in some ways astounding”

(Source-Participant 303-Service Provider).

#### Social isolation

Service users commonly described feelings of loneliness and disconnection from social supports, as this service user highlights:

“I don’t like Christmas…Christmas is for kids, for family, but for people like me it’s a trigger because you’re lonely…I am living on my own, I don’t have friends”

(Source: Participant 100044-Service User).

Among those who did have friends or family, some expressed reluctance to involve them in their mental health challenges.

“…depression is so huge like it’s such a huge problem and people don’t even like know… for me in my life like I was a makeup artist, I was very popular I have so many friends, I have so many colleagues and I am such a positive person like nobody would ever, ever, ever and even the very few people that I told that I suffer with depression nobody would ever, ever guess that…”

(Source: Participant 10001- Service User).

#### Personal growth and desire to contribute

Despite the significant challenges faced by service users, they also described being *proactive and self-navigating*. Several service users reported finding helpful resources on their own or through the help of friends. They discussed a desire to give back, and contribute their knowledge and lived experience to the community. Some described sharing information about resources they found helpful to other people in their community. Others expressed a desire to become peer mental health workers. Three had recently volunteered with community organizations (Participant 100016, Participant 100060, Participant 100098).

“[I] find information so I am really good at that and I have been able to find some resources that even some of the people that I have been working with, they are going oh I have never heard of that, so I go here you want a copy of that yeah, okay”

(Source: Participant 100098 –Service User).

This desire to give back drawing from their lived experience is often difficult to acknowledge and realize given that the mechanisms in place, such as supported employment, and suitable employment opportunities, such as peer support, are scant.

### Addressing mental health frequent ED user needs

Given the complexity of health and social challenges identified in this population, it is not surprising that perceived service needs were multifaceted, including *case management*, *therapy*, *recreation*, *and employment*. Certain interpersonal characteristics of case managers, including relatability, values-driven approaches, and a non-judgemental stance facilitated progress in addressing service needs and preferences.

#### Meeting complex health and social needs

Service users expressed a need for help with *tasks related to case management*, mostly centered around system navigation, intermediation, advocacy, and practical-needs assistance. *System navigation was seen as an important case management role*. While several service users were able to self-navigate, others required assistance. Both service users and providers commented on the difficulty of navigating the mental health system in a large urban centre. The system was seen as fragmented and cumbersome, so having professional support with knowledge of the system was an asset.

“[T]here is a lot of information for services but how do you get there and how do you know what you are looking for right? It, it helps to have someone who’s got some expertise in the field to help you navigate the system”

(Source: Participant 100016-Service User).

Participants also valued *peer support* as a potential means of system navigation, as this participants described (Source: Participant 100016 –Service User):

“It’s really great to have somebody who has been through a similar experience to yourself especially if it’s somebody coming into the hospital for the first time because it is really frightening and just have somebody disclose the fact that they themselves have experienced something similar and that things are going to be okay and to maybe even walk them through the process of what’s going to happen next…would be really great”

(Source: Participant 100016 –Service User).

An additional case management role identified was that of an intermediary *and advocate*, facilitating communication with other healthcare professionals. Often a service user would have multiple support providers, often not aware of each other, and the case managers would act to increase awareness and coordination of these supports. Case managers could also act as an intermediary between a service user and their family, facilitating needed communication in the midst of family conflict. With respect to advocacy, service users saw case managers as someone “on their side” (Source: Participant 100060-Service User), working in their best interests, in contrast to the stigma and discrimination they often perceived when accessing the healthcare system. Similarly, service providers talked about their role involving “time, stamina, stubbornness” (Source: Participant 305-Service Provider) to advocate for service users in the context of a healthcare system with numerous entry barriers.

In addition to system navigation, intermediation and advocacy, assistance with *practical needs was identified as a priority*. This could include anything from providing assistance with housing and legal issues to transportation tokens.

“The only way I can leave that building is if …I kill somebody or kill myself… Or I get a transfer into regular housing…which I can only afford up to $600 to $650 or whatever. So, I was just like okay I can do that right, not pleasant but I will do it right? So, we started looking into apartments and stuff like that”

(Source: Participant 100025 –Service User).

Service users also appreciated when case managers could meet them in the community and accompany them to appointments or help organize appointments. Certain *personal characteristics* of TCMs that enhanced their relationship with service users included being relatable, non-judgmental, and values-driven. When TCMs were perceived by service users as non-judgmental, they were more willing to communicate their problems and their history. Service users also appreciated TCMs whom they felt were passionate about their work and cared for their clients, as opposed to being “there for the pay cheque” (Source: Participant 100016-Servicer User).

#### Assisting/ encouraging personal growth

Service users identified that case managers facilitated their personal growth, including their *insight*, *their confidence*, *and self-management*. TCMs facilitated insight by helping service users draw connections between their mental health concerns, substance use, and environmental factors such as past histories of trauma. Some service users noted that these insights helped them feel more hopeful and empowered in their recovery trajectories. TCMs supported service user confidence building, encouraging assertiveness in interpersonal relationships, or facilitating participation in meaningful recreational activities, volunteering, and employment, giving them, as this service user described: “something to wake up for” (Source: Participant 100060-Service User).

“…my TCM taught me how to be strong, how to be a powerful woman, and to stand up for me and my rights… So if there’s something—like if he [her husband] says: “‘Can you make me a coffee?” and I don’t feel like making one, I say: “‘No, make it yourself… She gave me that power because I didn’t have that power before and I’m really grateful to her for that because I couldn’t stand up to him if my life depended on it”

(Source: Participant 100111-Service User).

Finally, some service users noted that their TCM had motivated them to begin taking more responsibility for addressing their needs, for example encouraging one service user to manage a poor housing situation (Source: Participant 100098-Service User).

This approach was also in-line with service user preference for addressing symptoms and achieving personal growth. Most service users desired some form of *therapy/counseling*, commonly articulated as “someone to talk to”, but they believed that talking to a psychiatrist would label them as mentally ill. This was an important issue given that they saw their problems as inherent to stressful life circumstances that they wanted to talk through, and not a psychiatric illness. Having the TCM assist with personal growth was also a way of circumventing negative perceptions about the medical system’s willingness to support and validate their concerns, due to pervasive stigma and discrimination.

“Because she [the TCM] wasn’t like a psychiatrist I didn’t feel like I was sick and going to someone to talk about my problems with and I felt like she was just someone there to help get me resources or just figure things out”

(Source: Participant 100026 –Service User).

#### Increasing perceived social support

Service providers talked about the idea of amiably relating to service users, almost like friends, while still trying to maintain professional boundaries. This could involve, for example, the use of informal language, humour, and a focus on strengths and interests.

“What would you be interested in if you had your choices in life…did you guys ever think about taking some university courses, you guys are retired, they’re free. These are the things friends talk to each other about and so in that way I am their friend…”

(Source: Participant 303-Service Provider).

Indeed service users saw their TCM as a source of *social support*. As one service user stated, “So now we’re getting to be, like, friends…” (Source: Participant 100087-Service User). For some service users who perceived that they had no one in their lives to rely on, this was a highly valuable relationship. TCMs were available to talk to service users, to visit them in hospital, and to meet with them in person and by phone at scheduled intervals. Some service users felt that when the TCM cared about their well-being, this could be a motivating factor for change, as this service user describes:

“…nobody cares, nobody cares, nobody cares… it’s just like there’s somebody right there in your face that cares, and you don’t even notice and it’s just like it dawned on me it’s just like hello you know, and then [the TCM] was there like every once a week, like bloody clock work…”

(Source: Participant 100025- Service User).

In one instance a service user referred to being authentically known by their TCM as a motivating factor for their recovery.

“I thought a lot about her [TCM] when I was in the hospital for four days… I thought she wouldn’t want to see me like this. She knows I’m stronger than this. She knows that drinking is killing me, you know. It was a good positive feeling that I got and I knew I had to fight for it”

(Source Participant 100111-Service User).

## Discussion

The goal of this study was to expose the complexity of needs of frequent ED users with mental health and addictions challenges in a large urban centre, and identify perceived key service ingredients addressing service needs and preferences of this population. The present study adds to the small but growing literature on frequent ED users’ own perceptions of their needs and life circumstances and on service user and providers’ experience of helpful case management ingredients [12, 13 15, 18].

In this study, frequent ED service users described experiencing multiple health and social needs, a clinical profile previously described in the literature [2,3]. They also frequently reported social isolation and histories of trauma. Trauma exposure and social isolation have both been associated with greater healthcare utilization and costs [4, 19–23]. Frequent ED users in this sample often connected their health concerns to past traumatic events, a finding that has been noted in other qualitative samples of frequent ED users [24]. Given the prevalence of psychological trauma and social isolation in this population, grounding interventions in trauma informed principles and an emphasis on building formal and informal social supports are important considerations in the provision of case management and ongoing health and mental health care.

Both frequent ED users and service providers in our sample described pervasive stigma and discrimination in healthcare encounters. Stigmatization of service users with mental illness by healthcare professionals is increasingly recognized as a problem, as stigma and discrimination have been linked with negative physical and mental health outcomes [13, 25–29]. Although healthcare professionals’ attitudes towards this population may be explained in part by the commonly held view that ED visits by the frequent user population are inappropriate [2, 3, 5], evidence suggests that frequent ED users generally have higher acuity health concerns and are more likely to be hospitalized compared to non-frequent ED users [2]. The incorporation of interventions that address stigma and discrimination, such as contact-based education, should be considered in future interventions involving people experiencing mental illness and addictions and frequent users of healthcare [30].

Given the multiple health and social needs of this population, calling for intervention by multiple providers and service sectors, it is not surprising that system navigation, advocacy, intermediation, and practical needs assistance were identified as key elements of interventions attempting to support them. Ontario, like many jurisdictions, has a fragmented, difficult to navigate health and social service system [31–33]. Breakdowns in communication and coordination between multiple providers can leave service users in receipt of patchwork care [33, 34], with primary care services feeling ill- prepared to address complex needs and navigate ever changing community-based services [33, 35]. Case managers are uniquely placed to act as system navigators, advocates, and intermediaries given their mandates to work with both formal services and informal supports [36]. In our study, participants identified certain helpful TCM interpersonal characteristics, including being relatable and non-judgmental. A warm interpersonal manner and a non-judgmental stance are recognized aspects of a strong working alliance [37, 38]. Service users have previously reported appreciating the use of humor by health care professionals, as well as friendliness, genuine interest, empathy, careful listening, and open mindedness [39]. Interestingly, in our study, service users saw TCMs as “friends” and a means of social support. Informal, flexible, friendship-like aspects of working relationships have been identified by service users in the literature as valuable [40, 41]. More research is needed to elucidate the benefits and risks of this informal relational style, including the risk for boundary crossing and unmet expectations [40].

Service user participants further valued TCMs who were seen to promote recovery and personal growth. Mental health services have been increasingly adopting recovery-oriented practices [42–46], although this is an ongoing system goal across many jurisdictions. In particular, service user participants’ expressed the desire to give back to their community, through volunteering and employment, and interest in training as peer mental health worker—opportunities not widely available in many settings. There has been an exponential growth in the use of peer support for patients with mental illness in recent years [47], offering numerous benefits for peer providers, such as increased sense of empowerment and financial independence, improved confidence and communication skills, and personal growth through self-knowledge, perseverance and initiative [47–49]. Supporting involvement in volunteering, training and employment initiatives are important components of helpful interventions.

## Strengths & Limitations

The involvement of a peer researcher is an important strength of this study, which has the potential to inform program planning in many jurisdictions facing similar challenges. Peer researcher involvement allowed for greater comfort and engagement of service user participants and confidence in the findings [50]. The study nonetheless has some limitations. Recruiting participants from a single large metropolitan city may limit generalizability. However, our participants were drawn from several settings and hospitals serving distinct districts of the city. Another limitation is the lack of validation sessions to confirm the interpretation of emerging themes with service users, though validation sessions were done with service providers. The findings, however, align with those previously reported in the literature and add to the knowledge base in a priority area for service improvement. A third study limitation was that service user participants recruited from general acute care hospitals were not differentiated from service user participants recruited from the specialized psychiatric facility. As our study participants, similar to frequent ED users in other settings [2] presented to multiple EDs, there was considerable overlap between those two groups of participants, preventing a more nuanced understanding of the needs and preferences of those presenting to secondary versus tertiary care facilities.

## Conclusions

Mental health and addictions frequent ED users describe experiencing multiple mental and physical health challenges, social isolation, and experiences of stigmatization when interacting with healthcare professionals. Helpful components of interventions targeting this population may include system navigation, advocacy, intermediation, and practical needs assistance. Valued characteristics of service providers include relatability, a non-judgmental stance, and a recovery orientation. Our findings may be helpful in future efforts to address the needs of this population.

## Supporting Information

S1 File CATCH-ED Questionnaires. (DOCX)
