# Supplementary material for: Perceived Case Management Needs and Service Preferences of Frequent Emergency Department Users: Lessons Learned in a Large Urban Centre
Source: PLoS One. 2016 Dec 21;11(12):e0168782. doi: 10.1371/journal.pone.0168782 (PMC5176181; doi:10.1371/journal.pone.0168782)
Supplement: S1 File — (DOCX) [file pone.0168782.s001.docx]

**CATCH-ED**

**Client Interview Guide**

*Welcome.*

*I am a researcher with St. Michael's Hospital. You have been asked to participate in this interview because you have been a client of CATCH-ED.*

*As you know, CATCH-ED is a program that works to connect people to health care and other services. People are eligible to become clients of this program if they visited the Emergency Department 5 or more times in the past year, at least once for a mental health or substance use concern.*

*I will be asking you a number of questions about the health and social services you use, and what you think about CATCH-ED. Your responses will not be shared with any of your service providers, including your Transitional Case Manager. I want to urge you to speak as openly and honestly as you are comfortable with.*

*I want to give you an opportunity to read through the consent form and to ask me questions.*

*[Participant reads the consent form or is read the consent form by the interviewer. Participant signs the consent form]*

*Please remember that participation in the interview is completely voluntary and that you can choose to stop the interview at any time. You are free to skip any question. Your interview will last from 60 to 75 minutes. If you wish to take a break, you may interrupt at any time. Nothing you say will be associated in any way with your name – it will all remain completely confidential. Again, I want to reassure you that your Transitional Case Manager and your other service providers will not know any of your responses.*

*To remind you, some of the questions I will be asking you are of a personal nature and you may feel uncomfortable answering them. Again, please feel free to pass on any questions you prefer not to answer.*

*Just to remind you, we would like to audio record the interview so that we don’t miss any of your answers. Is that still ok with you?*

*Do you have any questions before we begin?*

*[Interviewer responds to questions]*

*So let’s begin. [turn on recorder]*

*I’m with participant # _____________.*

**CATCH-ED – Previous Experience and Services**

*To begin, let’s talk a little about your life and the services you had before you were connected to CATCH-ED.*

1. Could you tell me a little about your life before you were connected to your Transitional Case Manager, and the CATCH-ED program?
   1. Can you tell me about what your mental health was like? Your physical health? About your use of alcohol or drugs?
   2. Where/to whom did you go for help or support? (egs. Service providers, friends, family members, etc.)
   3. What services and supports were you connected to before? (By services, we mean health services, such as a physician, nurse or psychiatrist; and social services, such as a counselor, a social worker, long-term case management. By supports we mean peer support, income supports, education or job supports, housing, court services, or any other supports you had). **Which of these services and supports are you still connected to?**
   4. What kinds of health care and social services were you missing?
   5. **Thinking about the other programs you are connected to now, or have used in the past, how is CATCH-ED different? What is helpful about those services? What is unhelpful about those services**?

**Client Experiences of CATCH-ED**

*Now I’d like to ask you about how you first connected with the CATCH-ED program, and what you were hoping it would offer you.*

1. How did you first get connected to the CATCH-ED program? Who referred you?

*Prompts*:

Can you tell me about your first visit with your Transitional Case Manager?

What stands out for you about that meeting?

1. What were you hoping CATCH-ED would help you with?

*Now I want to ask you about CATCH-ED did for you, and about the kinds of health care and social services you were connected to by your Transitional case Manager.*

1. What did CATCH-ED do for you?

*Prompts*:

What did your TCM do for you?

What health care or social services did your TCM refer you to?

*Health care:* Family doctor, psychiatrist, mh&a counseling, dental care?

*Social services:* long-term case management, income supports, education or job supports, housing, court services, anything else?

- 1. Which of these did you go to?
  2. Which of these did you find helpful? In what ways? Did these services and supports give you what you needed? In what ways? If not, what other services did you need?
  3. Which of these did you find unhelpful? In what ways?

What community resources or supports did your TCM connect you to? (e.g. peer support, recreational or leisure activities)?

1. Did you have any health or other problems that bothered you that CATCH-ED did not help you with?

*Prompts:* Physical health problems? Mental health problems? Alcohol or drug use problems? Other problems in your life?

- 1. What prevented you from addressing these problems?

*Prompts:* Your health, a lack of available services, wait times for services, distance/travel, you weren’t eligible, your level of interest, bad past experiences, not having reliable phone/mail/email access?

- 1. Did you talk to your TCM about this? Why or why not? What happened? Did you talk to anyone else about this?

1. Were there any service providers or services you wanted to visit or use more often, but couldn’t? (e.g. your TCM, the services the TCM connected them to)
   1. Which ones? Why?
   2. What got in the way of using this service more often?

**Key moment/Turning point**

1. Could you think back over the period when you were working with your Transitional Case Manager? Is there a key moment that stands out when things changed for you? For instance, was there a person, event or service that made the most difference? Could you describe this person, event or service? What happened?

**Changes in health status**

*These next few questions are about whether or not the CATCH-ED program has made a difference in your life.*

1. How would you describe your health now that you have received services from the CATCH-ED program?

*Prompts*: What has changed for you: Mental health/emotional health/physical health/alcohol or drug use;

Has your health improved, worsened or stayed the same?

**CATCH-ED – Reflection**

*Now I would like to talk about your thoughts and feelings about CATCH-ED.*

1. Would you say that CATCH-ED worked for you? If yes, in what ways? If no, can you say why not?

*Prompt*s: Did it make a difference in your life? Did it meet your needs?

1. Thinking about the CATCH-ED program overall, what was most helpful for you? Why?

*Prompts:* e.g. Services at the Community Health Centre (doctor, counselor, other)? TCM’s support? Peer support? Other services you were referred to? Anything else?

1. What about CATCH-ED was unhelpful for you?

*Prompts:* e.g. Service hours, duration of service period, level of support

*My last question about CATCH-ED are about whether or not you would recommend the service, and what you would change about it, if anything.*

1. Would you recommend the CATCH-ED service to someone with similar needs? Why would you recommend this service? Why would you not recommend this service?

*Prompt*: If you could re-design CATCH-ED, what would you do differently?

**Emergency Department Visits**

Finally, I’d like to ask you about your visits to the Emergency Department:

1. Since you started in the CATCH-ED program, do you visit the emergency department more, less or the same amount? Why?
2. Thinking about the last time you went to the ED, can you tell me what that was like?

Probes: What did you hope would happen? What actually happened?

1. When you go to the ED, what are the specific concerns that take you there? What are the specific reasons why you go?

Probes: Mental health concerns? Substance use? Physical Health? Does someone advise you to go? Are you transported there?

1. Can you describe how you feel about going to the ED?

Probes: Are there things you like about going to the ED? Could you please describe them? Are there things you don’t like? Could you please describe them?

1. What additional supports or services would affect how often you visit the Emergency Department?
2. Is there anything else that would affect how often you visit the Emergency Department?

**Conclusion – Wrap up**

1. Is there anything else you would like to add about your experiences in the CATCH-ED program?
2. Is there anything else you would like to add about your visits to the Emergency Department?

*Thank you very much for participating in the interview today. I appreciate your taking the time to share your experiences with me.*

**CATCH-ED Interview Guide**

**Service Providers**

*Thank you for attending this voluntary interview. The purpose of today's interview is to discuss the key program components of CATCH-ED. By components, we mean the critical ingredients of the services that are being delivered in the project. The interview will take about an hour.*

*Before we get started let’s review the consent form. Then you can decide if you want to participate in the interview.*

*[Interviewer reviews the information letter and consent form with the participant.]*

*What questions do you have before we begin?*

*[After questions have been asked and answered, the participant is asked to complete the consent form and give it to the interviewer.]*

*I am now going to start the audio recorder.*

*The purpose of today's interview is to discuss the key program components of CATCH-ED.*

**TCM Experiences of CATCH-ED**

1. Can you tell me about what it was like when you first became involved with the CATCH-ED program? What was your preparation like?
2. What is your role with CATCH-ED? Can you describe for me what you would do on a typical day? How does this differ from what you used to do/what you do for your non-CATCH clients? How is CATCH-ED’s way of working different from the ways you were used to working?
3. I’m wondering about the way CATCH-ED is organized and structured and how that works? Does it work well? Are there challenges?
4. What are the challenges in providing care for this population? Have you worked with clients with similar challenges before?
5. What about CATCH-ED makes it a good way to serve clients? What about it makes it more difficult to serve clients?
6. How has working with CATCH-ED changed your approach and management of the clients in this population?

*Probes:* Do you find it any easier or more difficult to manage these types of clients now? What has been the greatest advantage/disadvantage of the CATCH-ED approach?

1. How is care coordinated among members of the multi-disciplinary team (hospitals/CHCs/peer support/community mental health agencies)?

*Probes:*

Is coordination working? Why, or why not?

Can you describe the ways the hospitals/CHCs/MH agencies and peer support agencies work together?

What are your relationships like with:

ED hospital staff/Psych ED? Inpatient staff?

With Community Health Centre staff: Physicians? Mental Health and Addictions Counselors? intake workers?

**Description of the CATCH-ED Model**

1. What are the objectives of the CATCH-ED program, as you see them? That is, what do you think CATCH-ED sets out to do?
2. Do you think these objectives are being met? What makes it possible for these to be met?
3. Where have you seen trouble spots? What do you think has contributed to the development of these trouble spots?
4. What in your view are the most important components or ingredients of CATCH-ED (i.e. I’m wondering about the kinds of services and supports CATCH-ED offers) (TCM, CHC primary care, counseling, psychiatric support, other)?
5. What do CATCH-ED services do to help clients’ recovery?

What do you feel are the most helpful components of the intervention in facilitating client recovery? Do you think additional components are needed to facilitate client recovery?

1. (Are you familiar with the CTI model and CATCH-Homeless model on which CATCH-ED was originally based?) How are some of the components true to the CTI model? What is different? What is the same? What has been added? What has been learned in the process?

**Outcomes of the CATCH-ED Model**

1. What outcomes do you anticipate as a result of CATCH-ED? (e.g. improved health, reduced mental health and substance use crises, better quality of life.

*Probes*: Short term outcomes

Long-term outcomes

What are the anticipated timelines for these outcomes?

1. How do the different services and supports that CATCH-ED offers work to create these outcomes?

*Probes*: What specific activities (TCM, CHC primary care, counseling, psychiatric support, other) are central to producing these outcomes?

How do these components affect clients?

1. Do you think that additional components are needed to reduce emergency department visits? What are they?
2. What has been learned so far in the intervention about what is important regarding the services and supports needed for this population?
3. Overall, what do you think are the key factors that drive the success (or not) of the program?

*Probes*:

For example, the resources available, the relationships between the different project stakeholders, leadership, etc.

1. One of the objectives of the CATCH-ED program is to support clients to experience “Continuity of Care” which would mean that they are connected to appropriate community-based health and social services, and that their health and social service needs are met in a reasonably orderly and timely way, without interruptions.

What needs to be in place for this client group to experience continuity of care?

Improved quality of life?

Reduced use of acute care services?

1. If you had more time, what would you like to have done to further define or augment the intervention?

**Client Experience of CATCH-Model**

1. Why are clients using the ED frequently?
2. What were your clients told about the model? Can you tell me about what you think their expectations were from CATCH-ED?

*Probes***:** What did they say they wanted out of the program? Do you think anything surprised your clients about CATCH-ED? Do you think anything disappointed them?

1. What are the characteristics of participants who benefit most from CATCH ED? (such as mental health characteristics, cultural characteristics, substance users) What about the characteristics of clients who benefit least?
2. What changes, if any, did participants experience during the first 3-4 months in the program? Is this consistent with your expectations? Why or why not?
3. Can you talk about any changes you have noticed in how clients manage their care.

*Probes***:** Can you provide any examples as to a time when a client has responded differently to a problem or crisis as a result of receiving care through CATCH-ED (e.g. choosing not to go to the emergency department)?

1. What challenges and barriers have emerged as the project has been implemented? (e.g. referrals from the ED, training and supervision of TCMs, program structure, etc.)
2. To your knowledge, what are the barriers for participants who are not linked to CATCH ED (the usual care group) in accessing the services and supports they need?

*Probe:*

What is the quality of these supports?

1. Are there any other perceptions about the program you haven’t had a chance to mention you would like to add before we finish up?

*I am now shutting off the tape recorder.*

*Do you have any questions for me?*

*Thank you very much for your participation today. I appreciate your willingness to share your thoughts and opinions with me.*

**CATCH-ED Guide**

**CHC personnel**

*Welcome.*

**Description of the CATCH-ED Model**

1. Are you familiar with the objectives of the CATCH-ED? What are the objectives, as you see them? In other words, what is CATCH-ED trying to achieve?
2. Is CATCH-ED working to meet those objectives?
3. Where have you seen problems or trouble spots? What do you think has contributed to the development of these trouble spots?
4. Is it your impression that CATCH-ED referrals to your CHC have been low? Do you know why this is?
5. Could you talk about the services and supports that you provide your CATCH-ED patients/clients?
6. What are the services and supports that are available to CATCH-ED clients through your CHC? Primary care? Psychiatric consultation? Counseling? Other services? Are your patients accessing them? Why or why not?
7. Could you talk about the other services and supports that your patients have accessed through CATCH-ED (either directly through their transitional case managers or through other services and supports?)
8. What in your view are the (most important) components of the CATCH ED program (TCM, CHC primary care, counseling, psychiatric support, other)? Which services and supports are the most helpful?
9. What effect are these services and supports having on client outcomes and well-being?

How are CATCH-ED services and supports contributing to positive outcomes for your patients? For instance: improved health, reduced mental health and substance use crises, better quality of life, reduced use of acute care.

*Probes*:

What specific activities help produce these outcomes? (Examples: connection to other services, motivational interviewing, solution-focused therapy, etc.)

1. In terms of the multi-disciplinary approach to the intervention, could you describe the coordination between you and the TCMs? The coordination between you and the CHC counselor/physician? Is this working/not working?
2. Overall, what do you think are the key factors that drive the success of the program? (probes: for example, the resources available, the relationships between the different project stakeholders, leadership, etc.)
3. What are some of the factors that limit the success of the program? (e.g. system resources, the resources available, the relationships between the different project stakeholders, leadership)

**Outcomes**

1. What outcomes do you anticipate as a result of CATCH-ED?

Probes: Short term outcomes

Long-term outcomes

What are the anticipated timelines for these outcomes?

1. Do you think the intervention as it is delivered meets the needs of clients, and in what ways? Are there challenges in delivering the model?
   1. Are there additional services and supports that your patients/clients need that are not being provided through the intervention, but should be? What is missing in the CATCH-ED program that would help meet its objectives? Assist in your patients’/clients’ recovery?
   2. Do you think that additional components are needed to reduce emergency department visits? What are they?
2. What would you have done differently to define the intervention? To improve or augment the intervention?
3. Are there any other perceptions about the program you haven’t had a chance to mention you would like to add before we finish up?

*I am now shutting off the tape recorder. Do you have any questions for me? Thank you very much for your participation today. I appreciate your willingness to share your thoughts with me.*
